# Health Justice Partnerships: An International Comparison of Approaches to Employing Law to Promote Prevention and Health Equity

**DOI:** 10.1017/jme.2023.84

**Published:** 2023

**Authors:** Elizabeth Tobin-Tyler, Tessa Boyd-Caine, Hazel Genn, Nola M. Ries

**Affiliations:** 1: BROWN UNIVERSITY SCHOOL OF PUBLIC HEALTH AND ALPERT MEDICAL SCHOOL OF BROWN UNIVERSITY, PROVIDENCE, RI, USA; 2: HEALTH JUSTICE AUSTRALIA, SYDNEY, AUSTRALIA; 3: UNIVERSITY COLLEGE LONDON, UK; 4: UNIVERSITY OF TECHNOLOGY, SYDNEY, AUSTRALIA

**Keywords:** Health Justice Partnership, Medical-Legal Partnership, Health Inequities, Social Determinants of Health, Interprofessional Education and Practice

## Abstract

This article traces the development and growth of health justice partnerships (HJPs) in three countries: the United States, Australia and the United Kingdom.

## Introduction

Law is a powerful social driver of health^1^ and health inequities.^2^ As written, implemented and enforced, it plays a crucial role in structuring the distribution of resources across populations, the conditions in which people live, and inequitable treatment of marginalized populations based on race, ethnicity, indigenous status, disability, gender and socioeconomic status. Indeed, law can also serve as a critical tool for addressing health inequity through law reform, realization of human rights, and equitable enforcement of health-promoting policies. In recent years, there has been a growing body of legal scholarship focused on “health justice,” which provides a framework for identifying the role of law in structuring health inequities and envisions “health law as an instrument of social justice.”^3^ To achieve health justice, “the human rights, civil rights, value and dignity of all people [must be] acknowledged and actively fostered.”^4^

Recognizing the significance of the law’s role in health inequity, health justice partnerships (HJPs) have proliferated internationally, particularly in the US, Australia, and the UK. While HJP-like models exist in other countries, we highlight the US, Australia, and the UK because they have the longest history of HJP development and have all committed to national coordination and support for HJP programs in multiple sites and regions. HJPs — referred to as medical-legal partnerships (MLPs)^5^ in the US — train and partner health, social and legal service providers to explicitly identify, prevent and respond to violations of legal rights that harm health and well-being. In essence, HJPs put into practice the health justice framework that seeks to use law as a tool to remedy health inequity.^6^ HJPs also advocate for laws and policies that promote greater health equity. Although HJPs vary across countries due to differences in their health care, public health and legal systems, HJP leaders and practitioners from the US, UK, and Australia actively collaborate to share lessons learned and promote innovation in health, social, and legal care.

This article describes the conceptual framework underlying these partnerships; traces their development and compares their priorities, practices, approaches and challenges; describes the existing research base supporting their benefits; and highlights the role of interprofessional education and training in preparing a workforce that can practice effectively in HJPs. Throughout the article, we share lessons learned across countries regarding the benefits and challenges of HJPs and emphasize opportunities for international collaboration.

This article describes the conceptual framework underlying these partnerships; traces their development and compares their priorities, practices, approaches and challenges; describes the existing research base supporting their benefits; and highlights the role of interprofessional education and training in preparing a workforce that can practice effectively in HJPs. Throughout the article, we share lessons learned across countries regarding the benefits and challenges of HJPs and emphasize opportunities for international collaboration.

## The Conceptual Framework of Health Justice Partnership

The US, UK, and Australia all experience persistent health inequities. Government sponsored reports documenting population health status from the three countries point to significant disparities in health across populations associated with socioeconomic status, race, ethnicity, gender, and disability.^7^ Each country’s unique cultural, political, and economic context drives particular health inequities. For example, the history and legacy of slavery and Jim Crow laws in the US,^8^ vast socioeconomic health inequities in the UK despite universal health care coverage,^9^ and the ongoing impacts of colonization experienced by Aboriginal and Torres Strait Islander people in Australia,^10^ each propagates particular health injustices. The disparate effects of the COVID-19 pandemic on marginalized populations has further illuminated social and political drivers of health inequity in these countries.

Globally, social determinants of health (SDOH) research has propelled new strategies to address health inequities in public health, clinical medicine, and public policy. In the US, recognizing the effects of un- and under-enforced laws on the health of their patients, clinicians began formally partnering with legal advocates to address SDOH in the early 1990s through medical-legal partnerships.^11^ As described in greater detail below, in the UK and Australia collaboration across legal, health and social service sectors is not new, but formalizing these collaborations under the banner of “health justice partnership” started in Australia in 2012^12^ and the UK in 2016.^13^

### Common Assumptions of HJPs

HJPs across these three countries derive from at least four common assumptions: (1) low-income and other marginalized groups experience worse health due to injustices that are both directly caused or exacerbated by and potentially remediable through law; (2) access to justice — especially through direct legal advice and support — is crucial for improving health and health equity; (3) because the same low-income and marginalized populations experiencing poor health also experience poor access to justice, formal partnerships among service providers working with these populations can facilitate both justice and better health equity; and (4) by collaborating, health and legal service providers are in a unique position to identify the downstream health effects of law and policy failures.

Critically, HJPs develop shared goals based on these assumptions. Thus, health justice partnerships provide an opportunity for legal and health partners to share accountability for and measure success based on the intersection of health and justice. Because HJPs share these common assumptions, yet work in very different contexts, collaboration across the three countries has been invaluable. As described below, international collaboration has focused on three areas: promulgating best practices, developing shared research strategies and agendas, and disseminating HJP-related curricula and training materials.

### HJPs Work on Multiple Levels

HJPs work on multiple levels to promote health justice and equity. *Direct legal assistance*: Together, clinical and legal service providers identify unmet social and legal needs and support individuals and families through legal, medical, and social care in order to improve health and well-being. *Training*: cross-training of clinical and legal team members builds capacity, knowledge, and skills to better support patient and client care and advocacy. Interprofessional education for law and health professions’ students develops a new workforce with a broader understanding of the intersection between law and health and prepared to work across disciplines in HJPs. *Practice transformation*: Legal and health partners work to change the cultures of health care and legal services systems to provide more comprehensive and responsive care to marginalized patients and populations. *Policy change*: HJP practitioners identify upstream law and policy failures and advocate for reforms.^14^

## Models Across Three Countries

While health, legal and welfare or social services have worked together in some form for decades in each country, the development of formal HJPs across multiple sites and geographic regions in each country, has evolved in different ways. Furthermore, as national coordination and leadership has taken hold–such as the National Center for Medical-Legal Partnership (NCMLP) and Health Justice Australia (HJA)–the ability to learn from international colleagues and to share lessons learned has continued to shape theory and practice.

### The Growth of HJPs in the US, Australia and the UK

In the US, medical-legal partnerships grew out of pediatric clinicians realizing that legal and system barriers prevented low-income children and their families from enjoying the same opportunities to be healthy as more privileged patients and families. For example, they found that they could do little to reduce preventable hospitalization among pediatric asthma patients until housing safety violations in rental housing were addressed.^15^ Growth was slow during the 1990s, but accelerated in the early 2000s when medical-legal partnership was recognized by media, the American Bar Association and the American Medical Association as a key strategy to address the social determinants of health.^16^ As medical-legal partnerships began to proliferate across the US, a National Center for Medical-Legal Partnership (NCMLP) was established in 2006 to provide support to the growing network.

In Australia, a landmark study in 2012 indicated that many people were experiencing legal problems affecting their health, but were unlikely to seek help for those problems, and when they did, were likely to ask a non-legal advisor, such as a health professional.^17^ This evidence prompted legal assistance sector leaders to undertake research into collaborative service approaches to improve how they reached people with unmet legal needs. They studied international approaches to health-harming legal needs, including medical-legal partnerships in the US. Informed by this evidence, Australian community legal services piloted HJPs and shared the lessons of these pilots through both legal assistance and healthcare networks. These pilots led to a national movement for health justice partnership in Australia and later the establishment of a national center of excellence for health justice partnership, Health Justice Australia, recommended by Australian practitioner-led research that had examined the impact of NCMLP in the US.^18^

In the UK, social welfare law advice and support has long been recognized as a “tool for resolving social problems with a legal dimension.”^19^ As in the US and Australia, collaboration between health services and social welfare legal services reaches back to the 1990s, but a series of reports in 2013 connecting the provision of such services with prevention and early intervention in ‘smart’ locations created renewed interest and focused attention on health justice partnership as a formalized approach to interdisciplinary problem-solving and service delivery.^20^

### Different Contexts Lead to Different Approaches

At its core, health justice partnership works through collaboration across otherwise-siloed health and legal assistance services, supporting common cohorts of clients. But it reflects a wide range of service approaches, correlating to different needs, contexts and intended outcomes. These might include clinic, co-location or outreach models, with varying degrees of partnering beyond the presence of legal assistance in a non-justice setting.^21^

There is no single model of HJP. It is best understood as a person-centered innovation supporting services to respond to people’s multiple, intersecting health, legal and social needs where traditional and siloed service approaches can have limited effect. In Australia for example, at least five different service models have been identified under the overarching concept of health justice partnership: partnerships, integrated services, outreach, service hubs and student clinics.^22^ In the UK, HJP centers around the provision of free legal support to address legal problems affecting health, avoiding escalation of problems that might lead to health issues, and preventing ill health from leading to socio-legal problems.^23^ In the US and Australia, this direct provision of legal help through trusted healthcare settings sits alongside service-level efforts to reshape health and legal assistance around community need and system-level objectives to reshape the environment in which health inequity is experienced.^24^

In each country, legal partners tend to be publicly funded. This enables access to free legal assistance while targeting people who are unlikely to have the capability to address their legal needs on their own. However, government funded legal services are chronically underfunded, requiring service providers to limit both the number of people they can help and the extent of the help they can provide.^25^ Thus, health justice partnership contributes to reducing the “justice gap,” namely the often vast number of people with legal needs that would otherwise go unresolved.^26^

On the healthcare side, the service partners reflect a broad cross-section of the healthcare system. Because, unlike the UK and Australia, the US does not have universal health insurance coverage and the healthcare system is highly fragmented, medical-legal partnerships are targeted toward clinical settings serving low-income and vulnerable populations, such as federally qualified community health centers (FQHCs), primary care clinics with large Medicaid patient populations, and specialty hospitals, such as children’s hospitals and Veteran Administration hospitals.^27^ In the UK, about two-thirds of HJP legal advice services are physically located within a health setting such as General Practitioner (GP) practices, mental health services, hospitals and various community health services. Others work with hospice services, long-term care homes, and social care services.^28^ In Australia, health justice partnerships extend across most parts of the healthcare system, including hospitals, health districts and Aboriginal and Torres Strait Islander community-controlled health services. Beyond traditional healthcare settings, local government maternal and child health services and mental health charities are also involved.^29^

Health justice partnership is marked by ‘complexity and variation’, both within each domestic context and by comparison internationally.^30^ This reflects the different structural factors that shape the experiences of unmet legal need and health inequity in each country. In the US activities include screening referral, screening, case management, delivery of services and interprofessional and cross-disciplinary training and team meetings, together with policy change.^31^ Australia’s approach mirrors the US^32^ with additional activity such as secondary consultation^33^ and variation in the extent of work towards policy change.^34^ In the UK, co-location and referral pathways are common but the extent to which services are fully integrated or work jointly varies.^35^

The sharing of experiences across countries about the challenges and benefits of different approaches has been vital to improving practice. HJP leaders from the US traveled to Australia in 2012 to meet with multiple stakeholders to share lessons learned; Australian leaders have attended the NCMLP summits in the US; and two convenings have been held in the UK to bring together HJP leaders and practitioners from the UK, the US and Australia to share best practices for service delivery, funding and research.

### Challenges of Partnering Across Sectors

While interprofessional collaboration is core to the strategy of tackling complex problems, working in partnership also brings its challenges. Bringing together the approaches of healthcare and legal assistance requires navigating different professional approaches and lenses. For instance, a person seeking help for family violence might encounter very different responses from a healthcare professional, trained to view the problem in terms of health and safety, compared with a lawyer who will likely focus predominantly on rights.

Professional approaches also vary according to professional culture and ethical values and obligations. Lawyers are trained to advocate zealously for a client’s rights under the law while healthcare workers focus on diagnosing and treating illness, often as part of a team to coordinate care. Information-sharing in HJPs can be particularly challenging. For example, lawyers have strict ethical codes which prioritize confidentiality under attorney-client privilege, while healthcare workers may have mandatory reporting or other responsibilities predicated on shared access to information. Yet, HJPs have developed strategies that allow information-sharing and communication across professions, while still honoring ethical obligation. Building and maintaining partnership takes time and effort, forging relationships among professionals otherwise unaccustomed to working together.^36^ The cross-country collaboration has provided many opportunities for HJP leaders and practitioners to share strategies and tools, such as draft Memoranda of Understanding and interprofessional training materials that promote communication and understanding about roles and responsibilities among HJP participants.

These early planning and relationship-building activities are essential for developing the trust and confidence that support effective partnerships, but oftentimes funders expect immediate outcomes without acknowledging and valuing the longer-term work of culture change. In Australia, health justice partnership has been marked by short-term and uncertain funding, predominantly from justice-sector funders.^37^ In the UK, funding comes largely from charities and local authorities and much of it is short-term.^38^ However, recently there has been increasing local funding investment and interest from the UK Ministry of Justice. Australian and US HJPs have also relied on unsustained funding from philanthropy. HJPs in all three countries depend on significant in-kind support from health partners, such as physical space, administrative support and coordination of services.^39^

Notably, in the US half of all medical-legal partnerships report some level of financial contribution from their healthcare partner. These healthcare partners include hospital systems, FQHCs, Veterans Affairs hospitals and other clinics.^40^ Some of this support has been enabled by major federal legislation, the Patient Protection and Affordable Care Act (ACA) passed in 2010, which provided new incentives and funding for better care coordination and attention to non-medical needs in care settings.^41^ Specifically, the ACA promoted accountable care organizations which coordinate care among a group of health care providers (e.g. primary care, specialists and hospitals) using value-based payment mechanisms such as bundled payments or capitation. ACOs are incentivized to address upstream social factors in order to reduce downstream medical costs (such as emergency room utilization). With this greater focus on addressing SDOH and reducing unnecessary health care costs, healthcare systems have increasingly supported MLPs.^42^

Sustainability remains a key challenge across countries as HJPs grow and evolve rapidly and must respond to increasingly complex and intersecting health and legal needs. Despite contextual differences across countries, shared lessons learned have been crucial for funding strategies. For example, HJP leaders and practitioners have shared strategies for effective messaging about the benefits of investing in HJPs when communicating with skeptical health care and legal service organization administrators.

## Research Base Across Countries

The practitioner-driven development of HJPs in the UK, US and Australia demonstrates grass-roots legal and healthcare provider confidence in the value of the intervention as a tool for achieving justice and health equity. The intuitive logic of partnership and positive provider and user experiences have been important drivers of support for the movement. But this has largely proceeded ahead of a mature and systematic research base. While there is a relatively wide range of studies and reports addressing HJP models, practice and outcomes, there remains a need for targeted, rigorous research and evaluation evidencing the potential benefits of HJPs. Recent literature reviews provide important insights into the current state of the HJP research field, the evidence gaps, and the conceptual and methodological challenges in developing a research and evaluation program that would be internationally meaningful.

### What Have We Learned?

A comprehensive international literature search on HJPs (including academic, practice and gray literature) covering the period 1995-2018^43^ revealed a large number of studies predominantly from the UK and US. Strong evidence was found across all regions and service types for the effectiveness of HJPs in improving the socioeconomic circumstances of individuals (e.g., financial and housing security). There was also convincing evidence that HJPs reach patient groups most likely to be affected by health-harming legal needs who would otherwise not seek help for social welfare issues. This supports the contention that HJPs act on health and social inequities. The review found high quality studies (both quantitative and qualitative) showing improvements in patients’ mental health, particularly reduced stress, depression and anxiety, directly associated with legal interventions.^44^ However, an overall conclusion on the impacts of HJPs on individual health is more difficult to draw from published studies, given the diversity of patient populations and legal issues being addressed. Few studies internationally have used a control or comparison group to robustly assess changes in health, and studies taking an experimental approach have encountered significant methodological challenges.^45^

While research directly investigating the effectiveness of HJPs in preventing ill health was absent in the literature, there was good evidence for improvements in social determinants, including access to food, heating, and healthcare, and increased social participation, self-care, and self-confidence. Studies of the impact of HJPs on health service utilization showed inconsistent patterns and mostly lacked appropriate comparative evidence. Other impacts for health services and patient care had been explored to a lesser extent and were not the focus of much high-quality research. However, benefits identified qualitatively included supporting healthcare professionals to manage patients’ non-medical needs and improving both practitioner and patient experience. Catalyzing systemic change through legal and policy action has been more rarely reported in the literature, although there are case studies demonstrating the wide-reaching effects of these activities.^46^

A very recent review^47^ highlights the current evidence gap in evaluating the cost-effectiveness of adequate access to free legal services and proposes an interdisciplinary research agenda between health economics and legal-health services to address this gap. In an important development, the UK Ministry of Justice commissioned large-scale research on the impact of HJP and how to scale-up services. The work involved a robust impact, process and economic evaluation of existing co-located medical and legal services in general practice surgeries.^48^

In the US, NCMLP has undertaken comprehensive reviews of published research on HJPs. A 2013 review based on literature published between 1977 and 2012 found that the reviewed research was dominated by descriptions of the model and its variations, and very few studies provided systematically derived evidence of the benefit of HJP services on patients, provider institutions, and communities at large.^49^ Despite some limitations, the studies showed evidence of positive impacts of HJPs on, for example, patients’ stress levels, recovery of healthcare costs, financial return on investment, and education of providers. An update of the review published in 2020 drawing on additional studies found further evidence that HJPs significantly improved patient health and well-being, particularly relating to mental health, compliance with medical treatment, and reduction in Emergency Department visits.^50^ The 2020 review concludes that despite increasing attention given to HJP and the growing evidence base, more research is needed to demonstrate impact and to evaluate HJP service quality especially when scaling up.

In recent advancements: a US study demonstrated a reduction in hospitalization rates for children receiving legal interventions through HJP to address acute legal needs such as threat of eviction;^51^ and a further qualitative study of pediatric care suggests HJP can improve patient and population health by educating health providers about how to help patients with health-harming legal needs and social determinants of health.^52^

Health Justice Australia has made a significant contribution through describing and mapping the number and range of HJPs in Australia,^53^ and, with a broader international focus, has taken seriously the need to make progress on articulating a serviceable theory of change that will support the development of evaluation frameworks.^54^ A recent paper suggests the adoption of a common framework for measuring HJP/MLP outcomes.^55^ Proposing the concept of ‘client wellbeing’ as an outcome for health justice partnership, Forell argues that it aligns with a SDOH public health approach and highlights the broader societal value of legal assistance for low-income groups.

### Research Gaps, Opportunities and Challenges

HJP is a multifaceted intervention with a wide range of potential impacts. The reviews reveal diverse studies of variable scope, methods and quality, which indicate individual, community and system benefits. However, further work is needed to generate evidence in areas where there are gaps, and to produce further high-quality research that can strengthen existing knowledge. There are several challenges associated with the evaluation of HJPs across countries and systems. First, it can be difficult to structure research studies that evaluate the short, medium, and longer-term outcomes of HJP associated with resolution of particular legal problems, benefits to health and well-being, service and system change, and individual and population level impact, in different political and social contexts and systems. Second, it is quite complex to identify appropriate and consistent measures across systems that have deep structural differences (e.g., universal free healthcare in the UK as compared with the market-based healthcare system in the US, and the mixed model in Australia). Third, designing robust studies that can isolate and attribute the impacts of HJP as distinct from the effects of other activities or influences is a challenge faced by all HJP researchers, regardless of country or context.

Despite these challenges, the field is now sufficiently well-established that research efforts should move away from pilot and observational studies and focus more sharply on strengthening the evidence through methodologies that demonstrate impact and justify the investment necessary for accelerated and sustainable development of the intervention. At a convening sponsored by the British Academy and Wellcome in London in February 2020, HJP leaders and practitioners from the UK, US, and Australia met to share research challenges and strategies and to begin development of an HJP research agenda. The COVID-19 pandemic interrupted this work, but international collaboration is being reignited.

## Training the Next Generation in HJP: Interprofessional Education

Interprofessional education and training are essential to provide the core knowledge, skills and attributes students need as preparation for working effectively in HJPs. Students require an understanding of the complex determinants of health and how they “often manifest in the form of legal needs.”^56^ They need skills in identifying these issues, building relationships, and providing trauma-informed care in an interprofessional team context.^57^ By learning systems thinking, students are able to analyze root causes of problems and become equipped to advocate for changes to laws and policies. Collapsing disciplinary silos in the educational context provides students with early opportunities for “interprofessional dialogue which emphasizes the professions’ shared core values (e.g., respect for the individual and a commitment to reason-based decision-making, professional judgment, and experience) and mutual concerns for patient/client safety.”^58^ Interprofessional contact during training provides an early opportunity to foster awareness, interest, and capabilities for future work in HJPs.

### HJP Education Models

Understanding HJP education models requires consideration of (1) the competencies expected of students on successful completion of their studies in relevant health and legal disciplines, and (2) the approaches to and desired outcomes of interprofessional education.

In the US, Australia, and UK, various regulatory authorities set out the requisite knowledge, skills, and abilities expected of graduating medical and law students, which they must demonstrate to enter professional practice.^59^ These core competencies provide a foundation for continuing development in practice. Across these jurisdictions, there are commonalities in the desired competencies for students in law and health disciplines that provide general preparation for working in HJPs. These include competencies in: communication; teamwork; diversity and inclusion; professionalism and ethics; and systems level thinking. Within these parameters, universities and other education providers have flexibility to provide a range of learning opportunities, including innovative interprofessional education and training initiatives.

Interprofessional education refers to situations in which students (or practitioners) “of two or more professions learn with, from and about each other to improve collaboration and the quality of care and services.”^60^ Across the US, Australia, and UK, a variety of approaches are used, often in combination, including didactic classroom education, simulations, advocacy projects, and clinical and service learning.^61^ Classroom-based electives with cross-disciplinary enrollment may center on law as a social determinant of health, or focus on the intersecting legal and health needs of specific populations, such as children or older people.^62^ Simulations may involve students working in interprofessional teams to devise plans to assist a hypothetical client.^63^ Through involvement in advocacy projects, students gain experience in scrutinizing existing law and policy and may work in interprofessional teams to conduct research and write submissions to make a case for law reforms or policy change.^64^ International sharing of educational strategies occurs through traditional means, such as publications in peer-reviewed journals and conference presentations. In the US, NCMLP maintains a portal on its website which includes articles and other resources on topics that include curriculum and training *(https://medical-legalpartnership.org/resources/)*. As HJP educational programs continue to grow, opportunities will arise for formalizing international sharing and collaboration through a model such as the VIP Consortium (*vip-consortium.org)*, which is a global alliance of universities to support vertically-integrated projects, where students gain experience in large-scale multidisciplinary team projects.

Clinical and service learning provide hands-on learning opportunities for students in interprofessional team contexts. In the US, a growing number of university-based partnerships provide interprofessional training for medical, law and other students (e.g., nursing, public health) through clinical placements.^65^ Australian law schools often co-locate student clinics in community legal centers, which provides interprofessional training and exposure for students in law, social work, and other disciplines.^66^ In the UK, the University College London Integrated Legal Advice Clinic co-located with a general medical practice.^67^ Students gain experience with real-life client case matters and systemic advocacy projects may also be part of their clinical placements or internships with community organizations. Across all approaches to interprofessional education, students gain insights into health-justice intersections, build an understanding of professional roles and responsibilities, navigate ethical issues and acquire practical skills in issue identification, problem-solving, client and peer communication, and reflexive practice.^68^ While exposure to other professions through co-located models of service offer important interprofessional education opportunities, the ideal model for training the next generation of HJP leaders and practitioners is to educate and train students from different disciplines together–in the classroom and in the clinic.

The growth of HJPs across the US, UK, and Australia demonstrates both the increasing attention to the important role of law as a structural determinant of health and the value of partnerships between legal and health professionals to identify and address health inequities and injustices. International collaboration provides a rich opportunity to share lessons learned about approaches, practices, and challenges, further develop research agendas and methods, and promote innovative interprofessional education that cultivates and prepares health justice practitioners and scholars. HJPs offer enormous promise for addressing health injustice at multiple levels and across diverse settings and countries. International collaboration and scholarship will continue to be essential tools in expanding the reach of HJPs across the world, studying their benefits, and promoting best practices.

### Challenges in Interprofessional Health Justice Education

Interprofessional education is resource-intensive, requiring collaboration across traditionally siloed university faculties. Academic staff and clinical educators with the requisite knowledge, skills, persistence, and passion must work across disciplines to champion and sustain interprofessional initiatives. Relationships with external partners must be nurtured, particularly to provide sites for students to undertake interprofessional placements and internships under appropriate supervision. Specialized interprofessional electives must be squeezed into curricula crowded with core requirements and competing elective streams. These challenges need to be identified and addressed in the early stages of development before undertaking interprofessional educational initiatives in order to lay the groundwork for successful collaboration–just as careful planning and negotiation of professional roles is required for development of HJPs.

### Future Opportunities for Health Justice Education

Future opportunities for health justice education arise from (1) the burgeoning research evidence on HJPs, and (2) developments in education and practice that support social justice ambitions.

As discussed in the previous section, a growing body of research substantiates the benefits of HJPs in reaching people experiencing unmet legal needs and improving their socioeconomic circumstances. Knowledge of what works in HJPs and the outcomes they achieve can then be fed back to education and training settings to inform the work that occurs to prepare students for interprofessional practice. For example, in September 2022, NCMLP in the U.S. released an issue brief, *The Academic Medical-Legal Partnership: Training the Next Generation of Health and Legal Professionals to Work Together to Advance Health Justice,* which documented “how the academic medical-legal partnership (A-MLP) adheres to and deviates from” the core elements of medical-legal partnerships and provided insight into three components unique to academic MLP: 1) educating pre-professional learners, 2) intentionally creating interprofessional learning environments, and 3) contributing to the evidence base for the MLP model as a health equity intervention.^69^ In March 2023, the Solomon Center for Health Law & Policy at Yale Law School, the Georgetown Law Health Justice Alliance, and Penn State Dickinson Law School convened academic leaders from HJPs across the U.S. to share best practices, research, and interprofessional education strategies for academic HJPs.^70^ This convening can serve as a model for development of an international consortium that adopts best practices for academic HJPs, especially with regard to interprofessional training and education.

Studies on interprofessional education demonstrate desired impacts on students’ and trainees’ knowledge, attitudes, and behaviors.^71^ Notably, a 2021 systemic review concluded that HJPs are “an effective approach” to training on the complex determinants of health.^72^ Interprofessional learning experiences support students’ professional confidence and well-being. For example, law students who worked with health and social work students in a health justice clinic learned strategies “to better manage their own stress and mental health needs, which flowed on to providing a better service to the client.”^73^ The evidence base for interprofessional health justice education will be strengthened with more robust evaluation research design and longitudinal studies that follow people as they progress in their careers.^74^

Several trends in professional education and practice create a supportive context for health justice initiatives. There is growing emphasis on embedding experiential learning opportunities into university degree programs.^75^ Clinical placements and industry internships that provide interprofessional training offer added value. The growing number of high-quality resources to support partnerships means that new arrangements can be established more efficiently without having to “reinvent the wheel;”^76^ this can free up time and resources to develop and support student placements. In university curricula, greater focus is being placed on relational, cultural, and structural competencies and educating the next generation for social change.^77^ For example, the American Bar Association and the Association of American Medical Colleges have instituted training requirements and resources for student education on racism and bias.^78^ In Australia, university-wide initiatives are focusing on the Indigenization of curricula “to embed Indigenous histories, voices, experiences, knowledges, and ways of learning.”^79^

The unfolding crises of pandemics and climate disasters expose the inequities that divide and harm our societies. There is an urgent need for training the next generation of health justice leaders to pursue and implement transformative system changes. Continuing innovations in interprofessional education, training, and research provide the foundation for practices and structures that realize the promise of health justice partnerships.^80^

## Conclusion

The growth of HJPs across the US, UK, and Australia demonstrates both the increasing attention to the important role of law as a structural determinant of health and the value of partnerships between legal and health professionals to identify and address health inequities and injustices. International collaboration provides a rich opportunity to share lessons learned about approaches, practices, and challenges, further develop research agendas and methods, and promote innovative interprofessional education that cultivates and prepares health justice practitioners and scholars. HJPs offer enormous promise for addressing health injustice at multiple levels and across diverse settings and countries. International collaboration and scholarship will continue to be essential tools in expanding the reach of HJPs across the world, studying their benefits, and promoting best practices.
